# Supplementary material for: The water supply association analysis method in Shenzhen based on kmeans clustering discretization and apriori algorithm
Source: PLoS One. 2021 Aug 5;16(8):e0255684. doi: 10.1371/journal.pone.0255684 (PMC8341608; doi:10.1371/journal.pone.0255684)
Supplement: Attachment — Submitted filename: Reviewer Report.docx [file pone.0255684.s003.docx]

**Reviewer Report**

**Dear Editor**

Through my review of this round of scientific evaluation of the manuscript entitled “*The water supply association analysis method in Shenzhen based on kmeans clustering discretization and apriori algorithm*” and after reviewing the author's response to the comments of the reviewers. I found that the author has responded very well and submitted a satisfactory modified version that meets the research basics. The modified manuscript becomes more enhanced than before, and on this basis, Eventually, the author has adequately addressed the reviewer comments raised in a previous round of review and I feel the manuscript is now acceptable for publication. I here authorize the editor to accept it as my decision is to accept the research.

**Salih M Awadh**

**20 July 2021**
