# Supplementary material for: The water supply association analysis method in Shenzhen based on kmeans clustering discretization and apriori algorithm
Source: PLoS One. 2021 Aug 5;16(8):e0255684. doi: 10.1371/journal.pone.0255684 (PMC8341608; doi:10.1371/journal.pone.0255684)
Supplement: Attachment — Submitted filename: Reviewer comments.docx [file pone.0255684.s001.docx]

**Reviewer comments**

The manuscript entitled “The water supply association analysis method in Shenzhen based on kmeans clustering discretization and apriori algorithm” looks an applied research based mainly on the kmeans clustering and apriori algorithm that had been applied on Shenzhen, a city located to south of China. It was well structured and written in good way. However I recommend a major revision to address the following points:

***Abstract***

The research abstract seems almost vague as if it is a fragmented part of the research. The abstract should be reformulated to be easy to understand and expressive of the overall research content.

***Introduction***

I think the introduction was written in a format that could be described as good, but it still did not live up to the preferences. It is better to divide the introduction to include the scientific background of the main topic and the methods of work used in the research. After that, the research problem should be stated clearly and the reserch importance addressed. Then the previous studies in the field of research are listed. Accordingly, the introduction should be written in a more effective way to cover all aspects of the topic. The subject of the introduction focused on aspects and overlooked other important aspects. Some mathematical methods have been presented that can contribute to establishing a certain guess, while the water supply itself is not given any importance in terms of clarifying the factors controlling it in theory. The research problem should be clearly clarified in a separate sentence, and then presenting the originality of the idea and the importance of the research, what is the intended final benefit, and whether the research aims at any knowledge addition at the academic or applied research level. What makes the research subject to criticism is not to address more previous studies and present their most important conclusions. It is also preferable to redesign the target and write it in a clear text, away from ambiguity. I would prefer to re-state the aim of study “This study can establish valid SAR among features and recognize association degree of SAR. Finally, the value range of features in SAR can be obtained through the association rules”.

***Materials and methods***

- The situation of study area was included within the materials and methods section! It is not suitable to be placed in this section; it is better to be as an independent section.
- At last paragraph of the Materials and Methods, the author has stated “The data for this study came from the monthly data of Shenzhen Bureau of Water, the Shenzhen Bureau of Statistics, the Shenzhen Water Group and Digital Water System from 2004 to 2019. The methods in this study were developed in Python3”. This topic is not related directly to the study area but rather to the methods of work and data collection, so I would prefer to transfer it to suitable place.

What colors (green, orange, and blue) mean in Fig. 2.?

I do not understand why the author resorts to drawing equations for some common topics, and if he referred to computer software programs, it would be better, for example “spearman's rank correlation coefficient”.

The scatter diagrams need to be explained how clarifies the relationships between different features.

The expression 3D = 3 in section 2.2. Mining results and discussion: is not accepted to be written by this style, please.

The highest confidence degree in Table 3 is low (0.7)? Can such a result be taken into consideration?
